# Placental Pathology and Pregnancy Complications

**DOI:** 10.3390/jcm12155053

**Published:** 2023-08-01

**Authors:** John Kingdom, Jennifer A. Hutcheon, Sanne J. Gordijn, Dina El-Demellawy, David Grynspan

**Affiliations:** 1 Placenta Program, Maternal-Fetal Medicine Division, Department of Obstetrics & Gynaecology, Mount Sinai Hospital, University of Toronto, 600 University Avenue, Toronto, ON M5G 1X5, Canada; john.kingdom@sinaihealth.ca; 2 Department of Obstetrics and Gynaecology, University of British Columbia, 4500 Oak Street, Vancouver, BC V6H 2N1, Canada; jhutcheon@bcchr.ca; 3 Department of Obstetrics and Gynaecology, University Medical Center of Groningen, CB20, Hanzeplein 1, 9700 RB Groningen, The Netherlands; s.j.gordijn@umcg.nl; 4 Department of Pathology, Children’s Hospital of Eastern Ontario, Ottawa, ON K1H 8L1, Canada; deldemellawy@cheo.on.ca; 5 Faculty of Medicine, University of Ottawa, Ottawa, ON K1H 8M5, Canada; 6 Department of Pathology and Laboratory Medicine, Vernon Jubilee Hospital and University of British Columbia, Vancouver, BC V1T 5L2, Canada

Placental pathology assessment following delivery provides an opportunity to identify the presence and type of disease that can mediate major obstetrical complications, especially in cases where the fetus is growth-restricted, born premature, or stillborn, or if the mother suffers from severe hypertensive morbidities [1,2,3,4]. Placental pathology assessment enables many pregnancies complicated by fetal growth restriction (FGR) and/or hypertensive complications, including severe preeclampsia (PE), to be compartmentalized according to underlying mechanisms of placental disease vs. host risk factors, and then further divided according to placenta disease-specific pathways [5,6]. The most common of these is the spectrum of gross and histologic features of what is termed maternal vascular malperfusion (MVM). This has a distinct pathophysiology from fetal vascular malperfusion (FVM), which signifies obstruction of the feto-placental circulation [7]. Non-circulatory disorders, characterized by invasion of the placenta by the maternal innate immune system, are less common, but have potentially higher recurrence risks [8]. These include the invasion of the placental villi by T-cells, described as villitis of unknown etiology (VUE), and chronic histiocytic inter-villositis (CHIV), where large numbers of CD68-positive macrophages populate the inter-villous space [9,10]. Finally, and as sometimes co-exists with CHIV, the placental villi may become aggregated by a process of massive perivillous fibrinoid deposition (MPVFD) [11]. Uteroplacental perfusion is typically normal in all placental pathology types, with the exception of MVM disease; as such, these so-called “non-MVM” pathologies may be characterized by normal maternal blood pressure in the context of fetal growth restriction. Unexpected stillbirth may follow due to a lack of disease recognition by obstetricians. In cases where the fetus has been delivered vaginally, commonly following stillbirth, acute ascending bacterial infection may be layered on top of the underlying chronic disease, resulting in varying grades of chorioamnionitis, [10].

Knowledge of placental pathology findings is central to patient management in subsequent pregnancies, since these diseases have widely differing underlying causes and recurrence risks. For example, all features of the maternal metabolic syndrome, and cardiac risk factors in general, co-segregate with MVM disease [12]. Obstetrical complications associated with MVM disease of the placenta appear to be “acetylsalicylic acid (ASA) responsive” in future pregnancies, with a recent report demonstrating a 9% incidence of birth at <34 weeks in a subsequent pregnancy [13]. ASA prophylaxis, when initiated before 16 weeks’ gestation, substantially reduces the risk of preterm birth in association with severe preeclampsia, but has no independent effect on fetal growth [14]. Large-scale aspirin trials have not incorporated observations of placental pathology, presumably due to cost implications; therefore, any placenta-specific mechanism of action of aspirin to prevent severe preeclampsia is presently unknown. Vulnerability to recurrent MVM disease can be determined early in a future pregnancy using combinations of uterine artery Dopplers (to assess the development of uteroplacental blood flow) and the early profiles of circulating placenta growth factor, PlGF, in maternal blood [13,15].

Pregnancy screening for placental function may be incorporated into multi-modal screening for preeclampsia prevention at 11–13 weeks via aspirin prophylaxis [16]. However, later approaches to this concept of placental health testing have not, thus far, proven to be sufficiently accurate in the general pregnancy population. Given that the stillbirth rate in high-resource settings remains around 1/200, despite widely available ultrasounds, and over two-thirds are found to be growth-restricted with underlying placental diseases, there are considerable grounds for improvement. Combining high-quality ultrasound with measurement of PlGF (and possibly sFLT) at >28 weeks is presently under investigation in controlled clinical trials. In low-resource settings, where perinatal mortality is, sadly, far higher, attention to the quality of care at birth and to nutrition and infectious disease burdens offers a greater scope for improvement in care to reduce the risk of stillbirth. Nevertheless, the high rate of maternal death from severe preeclampsia and eclampsia—due to placenta MVM disease in low- and middle-income countries—suggests that angiogenic growth factor screening, if correctly deployed, could save lives through targeted preterm delivery in the maternal interest.

Much criticism has been leveled at the discipline of placental pathology, both for its accuracy between reporting physicians and for its mechanistic relevance to future care. The utility of placental pathology can be improved in two main ways. The first is via synoptic reporting, whereby specific gross and histologic findings are reported in a standard manner, with linkage to lab information systems [17]. The second important approach is to develop educational tools that equip clinicians, especially in maternal–fetal medicine, to be able to effectively communicate the implications of the pathologic findings to patients in ways that meaningfully improve both their inter-pregnancy and their future pregnancy care [17,18]. Revising the International Classification of Diseases (ICD) codes related to placental disease to better align with criteria used in existing synoptic reporting systems (Amsterdam criteria) could facilitate the accumulation of linked health data. This would facilitate the accumulation of linked health data that would be far more meaningful than at present, so as to further study the long-term health implications of producing a placenta that has one of these distinct pathologies. There is an important need for more population health data linking risks such as malnutrition, obesity [19], environmental exposures, and poverty with placental pathology and maternal and fetal health outcomes to inform patient counseling and public health policy.
